# Supplementary material for: Improving desirable agronomic traits of M2 lines on fourteen Ethiopian Sesame (Sesamum indicum L.) genotypes using Ethyl Methane Sulphonate (EMS)
Source: PLoS One. 2023 Sep 26;18(9):e0287246. doi: 10.1371/journal.pone.0287246 (PMC10522002; doi:10.1371/journal.pone.0287246)
Supplement: Attachment — Submitted filename: Response to Reviewers.docx [file pone.0287246.s002.docx]

**01.05.2023**

**RE:** Response to Reviewers

**MS Title:** Improving desirable agronomic traits at M2 lines of fourteen Ethiopian Sesame (*Sesamum indicum* L.) genotypes using Ethyl Methane Sulphonate (EMS)

**MS No:** PONE-D-22-29725

Dear Reviewers,

We would really appreciate and thank for your valuable comments/issues raised on the manuscript and were highly valuable to improve our manuscript. Accordingly, we have tried to justify some of your points, address some of the comments, and correct and modify other grammatical ang language problems as much as possible so as to improve the manuscript.

We are, hence, very hopeful that we have improved the manuscript to your satisfaction of all the points you raised.

The details of our responses to the comments/questions raised are given below under each comment.

**Reviewer #1:** I revised the manuscript entitled Improving desirable agronomic traits at M2 lines of fourteen Ethiopian Sesame (Sesamum indicum L.) genotypes using Ethyl Methane Sulphonate (EMS)".

**The Abstract**

Overall, the abstract provides a clear and concise overview of the study's purpose, methods, and results. The authors effectively highlight the importance of improving sesame yield in Ethiopia and describe how they used EMS to induce mutations in 14 sesame genotypes. They also provide a brief summary of the phenotypic changes observed in the M2 lines, and identify potential mutant genotypes that could have better desirable agronomic traits.

One strength of the abstract is that it clearly states the significance of the study, highlighting the urgent need to increase sesame yield in Ethiopia. Additionally, the authors effectively convey the novelty of their approach, noting that this is the first report on using EMS to generate desirable agronomic traits in Ethiopian sesame genotypes.

However, the abstract could benefit from additional information regarding the specific quantitative and qualitative agronomic traits that were measured and analyzed, as well as a more detailed explanation of the statistical analyses used to assess the significance of the observed phenotypic changes. Additionally, the authors could include more details about the potential implications of their findings and how they could inform future research in sesame improvement.

**Response:** Comments addressed. Corrected as per the given comments in ‘Tracked Change Formats’

Finally, the abstract could be strengthened by including more specific keywords that accurately reflect the content of the study, such as "mutagenesis" and "plant breeding." Overall, the abstract provides a solid summary of the study's main findings and contributions to the field, but could benefit from more detailed information and additional keywords.

**Response:** Comments taken. Corrected as per the given comments in ‘Tracked Change Formats’

**Introduction:**
Overall, the introduction provides a good overview of the significance of sesame as an oilseed crop and its various uses in nutrition, industry, and medicine. However, there are some issues with the presentation of information and the structure of the text.

Firstly, the introduction could benefit from a clearer and more concise thesis statement. While the general aim of the study is mentioned towards the end of the text, a more focused research question or objective would help to guide the reader and provide context for the subsequent discussion.

**Response:** Comments addressed. Changes have been made in ‘Tracked Change Formats’ as per the given comments.

Secondly, the introduction could be improved by providing more specific information about the challenges facing sesame production, such as the specific diseases and pests affecting the crop, and the current state of breeding efforts to address these challenges.

**Response:** Major diseases and insect-pests that affect sesame production are described towards the end of paragraph one of the introduction part. However, since the main objective of this study is to produce mutants with better agronomic traits, the authors focused only on the detailed findings conducted on induced mutation in relation to the genetic architecture of the crop.

Additionally, the use of excessive citation of sources in the introduction can make the text difficult to follow and distract from the main points being made. The author should consider condensing some of the information into a more coherent narrative and relying on a few key references to support their argument.

**Response:** Comments considered. Corrected as per the given comment in ‘Tracked Change Formats’

Finally, the language and grammar in some parts of the text could be improved for clarity and precision. For example, the sentence "Therefore, it is vital to reduce plant height significantly and enhance lodging resistance so as to increase sesame production using chemical mutagenesis" could be rewritten for better clarity and readability.

**Response:** We have rewritten the manuscript in more informative manner and have made significant grammatical and language corrections to improve the quality of the	manuscript.

**Methodology section:**

The material and methods section in this study appears to have some errors and critique issues. Firstly, the description of the seeds used in the study lacks information about the storage conditions, which could affect the seed quality and subsequently the results of the study.

**Response:** As the seeds were collected from Humera Agricultural Research Center, all the information about seed storage condition criteria were taken in to consideration by the research center.

Secondly, the method of sterilization is not adequately described, specifically the duration and temperature of the sulfuric acid and glacial acetic acid treatment. This can lead to inconsistent results due to differences in sterilization efficacy.

**Response:** This is a very valuable comment. We have mentioned some procedures/steps used in sterilization of the seeds. But all the steps are not mentioned as we have cited two references /number 25 and 27/ to indicate that we have followed the procedures used by them.

Moreover, the section on treatment with EMS lacks information on the rationale behind the choice of EMS concentrations used, and the reason for using Sörenson phosphate buffer and H3PO4.

**Response:** EMS mutagenesis of plants is a well-tested approach, which can induce point mutation in the DNA (genetic material) of plants altering gene expression and help to screen out favorable phenotypic expressions. Sörenson phosphate buffer was used in this study to adjust the PH of the solution. All the concerns raised are addressed in the previous researches cited in the manuscript (25, 28, and 29).

Additionally, the method of planting the treated and untreated seeds is not clearly described, and it is unclear whether the seeds were randomly assigned to the experimental plots.

**Response:** Comments considered. Corrected as per the given comment in ‘Tracked Change Formats’. Both the treated and untreated seeds of the genotypes were planted randomly.

The data collection and analysis method in this study appears to be appropriate; however, there is a lack of information on the method used for assessing the level of maturity of the plants before data collection.

**Response**: By maturity, the authors mean 75% maturity, which was identified based on the color of leaves and capsules of the plant.

Furthermore, the study did not include a description of the environmental conditions, which could impact the results of the experiment.

**Response:** Comments taken. Corrected as per the given comment in ‘Tracked Change Formats’.

In conclusion, the material and methods section in this study has some errors and critique issues that need to be addressed for the results to be more reliable and accurate.

**Response:** The authors have tried to significantly edit/modify the materials and methods considering the comments which are indicated in ‘Tracked Change Formats’.

**Results:**
The study conducted ANOVA to investigate the effects of EMS treatment on quantitative and qualitative agronomic traits of M2 lines of Ethiopian sesame genotypes. The results showed significant effects of genotypes, EMS concentrations, and their interaction on the quantitative traits including ground distance to first branch, internode length, leaf length, leaf width, and petiole length. However, some quantitative traits such as plant height, petiole length of basal and middle leaves showed non-significant effects at the interaction of genotypes and EMS concentration. The study found significant variations in the mean values of different agronomic traits among the tested genotypes, with some genotypes exhibiting higher or lower values for certain traits in response to EMS treatment. However, the study did not provide a detailed discussion on the mechanisms and possible factors influencing these variations.

**Response:** In the result part, we have simply interpreted the results but the mechanisms and possible factors influencing the variations are discussed in the discussion part**.**

Further studies are needed to investigate the underlying genetic and physiological mechanisms responsible for these variations and to develop effective strategies for improving the agronomic performance of sesame genotypes.

**Response:** True! Already addressed.

**Discussion**
The discussion section of this article provides an overview of the potential benefits of mutation breeding in improving the yield and desirable agronomic traits of sesame plants. However, the discussion lacks specificity and detail in certain areas. For example, while the article mentions the development and release of various mutant sesame varieties globally, it does not provide sufficient information on the specific methods and techniques used to develop these varieties.

**Response:** The authors have tried to improve the methods and techniques in the materials and methods considering the comments which are indicated in ‘Tracked Change Formats’.

Additionally, the article does not explore the potential limitations and drawbacks of using mutation breeding as a means of genetic improvement in sesame plants.

**Response:** Comments addressed

Furthermore, the article lacks clear research objectives and hypotheses. Although the authors report on the effects of EMS treatment on plant height and internode length, they do not articulate the specific research questions that they set out to answer through their investigation. Additionally, the article does not provide a clear explanation of the significance of the results obtained from this study or how they contribute to the broader field of sesame breeding research.

**Response:** The research objectives and hypotheses have already been described in the introduction part. The authors have tried to describe the various challenges/factors affecting sesame production globally, despite the alarmingly growing demand of oil. We also tried to justify the need/urgency of undertaking induced mutation on sesame so as to boost its production and productivity.

Overall, the article would benefit from a more focused and structured discussion section that clearly articulates the research questions and objectives, provides a detailed analysis of the results, and explores the potential implications and limitations of the findings.

**Response:** Significant modification has been made in the discussion part. Already shown in ‘Tracked change formats’ in the manuscript.

**Conclusion**
Overall, the conclusion of this study is well-written and provides a clear summary of the findings. However, there are a few areas where it could be improved.

Firstly, the conclusion could benefit from a more explicit statement about the significance of the findings. While it is stated that EMS is a powerful mutagen that produces desirable agronomic traits, the conclusion could be strengthened by explaining why this is important. For example, how might these findings be useful in breeding programs or in improving food security in Ethiopia?

Additionally, while the conclusion recommends further investigation and experimentation into mutant sesame genotypes, it could be more specific about what this should entail. For example, what specific traits or markers should be investigated, and what types of experiments should be conducted?

Finally, the conclusion could benefit from a brief discussion of the limitations of the study. For example, were there any factors that may have influenced the results or could have impacted the interpretation of the findings? Addressing these limitations would strengthen the conclusion by acknowledging the potential for future research to build upon these findings.

**Response:** We have tried to address the comments given for the conclusion as shown in ‘Tracked Change Formats’ in the manuscript.

**Recommendation section:**

There is a significant oversight in this study as it fails to include a recommendations section outlining possible interventions for future research. The absence of a recommendations section limits the usefulness and practical application of the study findings. It would be helpful to provide specific suggestions for future research on this topic, including potential areas of investigation and experimental designs that could be used to build on the current results.

Additionally, without recommendations for further research, the study's conclusions may seem incomplete or insufficiently supported by evidence. It is essential to address this shortcoming by providing a detailed recommendations section that can guide future research in this area.

**Response:** The recommendations are included at the end of the conclusion part.

**Reviewer #2:** Improving desirable agronomic traits at M2 lines of fourteen Ethiopian Sesame (Sesamum indicum L.) genotypes using Ethyl Methanesulphonate (EMS) seems an intersting study but it can be better if authors can add standaerror values in tables where mean values were used.

**Response:** Comments addressed. Standard error values are included as shown in ‘Tracked Changed Formats’.

Furthmore authors should write abstract in a quantitative way by firstly writing rationale then method and results in numbers. Recommendations to all stakeholders should also be given.

**Response:** Comments taken. Corrected as per the given comment in ‘Tracked Change Formats’.
