# Supplementary material for: Improving desirable agronomic traits of M2 lines on fourteen Ethiopian Sesame (Sesamum indicum L.) genotypes using Ethyl Methane Sulphonate (EMS)
Source: PLoS One. 2023 Sep 26;18(9):e0287246. doi: 10.1371/journal.pone.0287246 (PMC10522002; doi:10.1371/journal.pone.0287246)
Supplement: Attachment — Submitted filename: Reviewer comment-Improving desirable agronomic traits at M2 lines of fourteen Ethiopian Sesame.docx [file pone.0287246.s003.docx]

Reviewer comment-

Based on the errors and mistakes found in the manuscript, it is clear that the paper needs significant revisions and improvements before it can be considered for publication.

Firstly, there are several spelling and grammar mistakes that need to be corrected to improve the overall readability and coherence of the manuscript. Additionally, some of the sentences are unclear or convoluted, and the authors need to revise them to improve clarity.

Moreover, the conclusion section contains some inaccuracies and unsupported claims. For instance, the authors state that "the changes observed in both qualitative and quantitative traits of this study suggested EMS to be a powerful chemical mutagen that produces desirable agronomic traits", but this conclusion is not supported by the data presented in the paper. The authors need to provide a more nuanced and evidence-based conclusion.

Finally, the authors should provide more details about their experimental design and methods, as well as their statistical analyses. They should also provide more context and background information to help readers understand the significance of their findings.

In light of these issues, the paper cannot be accepted for publication in its current form. However, if the authors are willing to make the necessary revisions and improvements, the paper could be reconsidered for publication after undergoing a thorough peer-review process.

Please look the following errors and mistakes of your paper:

**Abstract**

Overall, the abstract appears to be well-written and structured. However, a few minor improvements could be made for clarity and precision.

1. The first sentence could be revised to be more concise: "Sesame is an important oilseed crop cultivated in Ethiopia as a cash crop for smallholder farmers."
2. "High and stable yield is urgent and a requisite to achieve the global oil demand" - This statement is unclear and could be rephrased to better convey the intended message.
3. "Chemical mutagens" could be defined or explained for readers who may not be familiar with the term.
4. The use of "EMS-treated genotypes" could be simplified by stating "mutant genotypes generated by EMS treatment."
5. The sentence "Mutants obtained from Baha Necho, Setit 3 and Zeri Tesfay were identified as potential mutants for better desirable agronomic traits" could be rephrased for clarity. For example: "Baha Necho, Setit 3, and Zeri Tesfay showed the most promising changes in desirable agronomic traits."
6. "Quantitative and qualitative data were recorded and analyzed using analysis of variance with GenStat 16 software" - This sentence could be improved by stating the specific quantitative and qualitative traits measured in the study.
7. The abstract could benefit from a statement about the significance of the findings and their potential applications in the field of sesame cultivation.

**Introduction:**

there are a few errors and scholarly writing mistakes that should be corrected:

1. In the first sentence, the phrase "widely cultivated all around the world" is redundant, as "widely cultivated" already implies global distribution.
2. In the second sentence, "high-quality" should be hyphenated to indicate that it is a compound modifier.
3. In the third sentence, "digestible fiber" should be "dietary fiber," as not all fiber is digestible.
4. In the fourth sentence, the phrase "industrial and pharmacological uses" should be "industrial and pharmaceutical uses," as "pharmacological" refers specifically to drug-related uses.
5. In the fifth sentence, the phrase "polyunsaturated fatty acids, phytosterols, tocopherols and unique classes of lignans such as sesamolin and sesamin" should be separated by commas to make it easier to read.
6. In the seventh sentence, the phrase "indeterminate growth pattern" should be "indeterminate growth habit."
7. In the eighth sentence, "low yielding capacity" should be "low yield potential."
8. In the ninth sentence, "seed retention" should be "pod shattering resistance."
9. In the eleventh sentence, "Sesamum indicum L." should be italicized, as it is a scientific name.
10. In the twelfth sentence, "facilitate mechanized harvest" should be "facilitate mechanical harvesting."
11. In the thirteenth sentence, "M2 lines" should be explained, as it may not be clear to all readers what this term means.
12. Finally, the last sentence could be revised to read: "The main objective of this study was to use EMS to induce desirable agronomic traits in 14 Ethiopian sesame genotypes, with the aim of accelerating breeding programs and improving the crop's productivity."

**Methodology:**

some errors and improvements can be made:

1. The units for altitude in Table 1 are missing. The authors should specify if it is in meters or feet.
2. The source of the data in Table 1 is not cited correctly. The authors should provide the full citation for the "MoARD, crop variety register book 2010- 2017".
3. In the methodology section, it is unclear what the abbreviation "ARC" stands for. The authors should define all abbreviations used in the article.
4. The methodology section should include more information on the statistical analysis performed, such as the type of statistical test used and the level of significance.
5. There is no information provided on the type of plant species and the number of plants used for each treatment.
6. In the section describing the recorded quantitative data, there are no units provided for the measurements. The authors should specify the units used for each measurement.
7. There are some minor formatting errors, such as inconsistent capitalization and inconsistent use of abbreviations.
8. The authors should consider providing more information on the potential risks and limitations of using EMS as a mutagen.

**Result**

there are some errors and areas for improvement, including:

1. In the first paragraph, the phrase "including ground distance to first branch (GDFB), internodes length (IL), length of top leaf (LTL), width of basal leaf (WBL), width of top leaf (WTL), length of marginal leaf (LML), width of marginal leaf (WML), and petiole length of top leaf (PLTL)" is quite long and could be difficult for readers to parse. Consider breaking it up into smaller, more manageable pieces.
2. In Table 3, there is an error in the "df" column for the "Treatment" row - it should be "1" instead of "0".
3. Also in Table 3, the "Variety" and "Treatment" rows should be switched in order, so that the row for "Treatment" comes first.
4. In Table 4, it would be helpful to include units for the different traits (e.g. cm for length measurements).
5. The phrase "the least ground distance was recorded in the treated genotypes including Setit 1 and Setit 3 (7.67 and 8.00) cm, respectively" is a bit awkwardly phrased - consider rewording to something like "the treated genotypes with the smallest ground distance to first branch were Setit 1 (7.67 cm) and Setit 3 (8.00 cm)."
6. It would be helpful to define any abbreviations or acronyms used (e.g. ANOVA, EMS) before using them in the text.
7. In the first sentence of the Results section, "many changes" is too vague and imprecise. It would be better to specify what kind of changes were observed.
8. The sentence "On the other hand, the analysis indicated that application of EMS didn’t affect the other agronomic traits such plant growth type, branching pattern, stem shape in cross section, leaf color, leaf hairiness, and leaf arrangement in all the genotypes (Table 5)" is poorly constructed and confusing. A better way to write this would be: "However, the application of EMS did not result in any significant changes in plant growth type, branching pattern, stem shape in cross section, leaf color, leaf hairiness, or leaf arrangement in any of the genotypes tested (Table 5)."
9. In Table 5, the meaning of some of the abbreviations is unclear. For example, "MSC" and "SH" are not defined.
10. The sentence "EMS treatment of the seeds caused changes in basal leaf profile in ADI, ACC44, Setit 1, Setit 3, and Gondar 1 genotypes from absent to weak" is unclear. It is not clear what is meant by "absent" and "weak" in this context.
11. There are some grammatical errors throughout the text, such as missing articles ("the", "a"), inconsistent use of verb tense, and incorrect subject-verb agreement. These errors do not significantly detract from the clarity of the writing, but they could be corrected to improve the quality of the article.

**Discussion:**

1. The sentence "Mutation breeding has hence been one of the breeding strategies used to minimize the aforementioned yield reducing factors [38, 39 As a result, more than 147 important sesame mutants associated with leaf, capsule, male sterility, flower, disease resistance, maturity and other agronomic traits were reported globally [33-35]" is confusing and incomplete. It would be better to split it into two sentences and clarify the relationship between the sources cited. For example: "Mutation breeding has been one of the breeding strategies used to minimize yield-reducing factors in sesame [38, 39]. As a result, more than 147 sesame mutants associated with various agronomic traits, such as leaf, capsule, male sterility, flower, disease resistance, and maturity, have been reported globally [33-35]."
2. The sentence "In Ethiopia however, yield remained very low due to a diverse set of factors including shattering capsule, determinate growth habit, branching, reducing plant height, diseases and insect pests, drought, waterlogging, salinity, and lodging." contains a list of factors that are not parallel in structure. It would be better to revise it as follows: "In Ethiopia, however, yield remained very low due to a diverse set of factors, including shattering capsules, determinate growth habit, branching, reduced plant height, diseases, insect pests, drought, waterlogging, salinity, and lodging."
3. The sentence "All these findings, hence, suggest that mutation breeding is crucial to produce genotypes with significant desirable traits such as determinate growth habit, shattering resistant capsules, synchronous (uniform) flowering and homogeneous maturity in a short period of time" is too long and complex. It would be better to split it into two sentences and simplify the wording. For example: "These findings suggest that mutation breeding is crucial for producing sesame genotypes with desirable traits, such as determinate growth habit, shattering-resistant capsules, synchronous flowering, and homogeneous maturity in a short time."
4. The sentence "Moreover, the results of this study were in agreement with other previous studies, where plant height of mutated dw607 (dwf1) with EMS mutagenesis was significantly declined by more than 40% (from 176.00 to 118.25 cm) as compared to the wild type, Yuzhi 11 [16, 36]." contains too much information in one sentence. It would be better to split it into two sentences and clarify the relationship between the sources cited. For example: "Moreover, the results of this study were consistent with previous studies that have shown a significant reduction in plant height of mutated dw607 (dwf1) using EMS mutagenesis [16, 36]. In one study, the plant height was reduced by more than 40% (from 176.00 to 118.25 cm) compared to the wild type, Yuzhi 11."
5. The sentence "The dwarfing trait and short internode length have been also reported in mutant dw607 [16]." contains a grammatical error. It would be better to revise it as follows: "The dwarfing trait and short internode length have also been reported in the mutant dw607 [16]."
6. The sentence "From these, we can infer that these variations would be due to the artificial mutation..." is awkwardly phrased. It would be better to revise it as follows: "These findings suggest that the observed variations in sesame genotypes are due to the induced mutations."

**Conclusion:**

1. In the first sentence, it would be more accurate to state that the study analyzed the effect of EMS on improving desirable agronomic traits in M2 lines, rather than on the genotypes themselves.
2. The phrase "various changes" in the second sentence could be more specific and indicate which specific traits were affected by EMS treatment.
3. In the third sentence, it would be more appropriate to state that EMS is a powerful chemical mutagen that can potentially produce desirable agronomic traits, rather than definitively stating that it does so.
4. The phrase "lead to synchronous maturity" in the fourth sentence could be more precise and indicate how this would impact yield losses.
5. In the fifth sentence, it would be more appropriate to use the phrase "marker-assisted selection" instead of "different markers like SSR, RAD-Seq, SLAF-Seq, SNPs". These techniques are used to identify genetic markers associated with desired traits, which can then be used to select plants with those traits.
6. The use of the word "however" in the final sentence is somewhat awkward and does not add much to the sentence. It could be omitted without affecting the meaning.
